# Unveiling novel drug-target couples: an empowered automated pipeline for enhanced virtual screening using AutoDock Vina

**DOI:** 10.1093/bioadv/vbaf267

**Published:** 2025-11-12

**Authors:** Sveva Bonomi, Stefano Carsi, Emily Samuela Turilli-Ghisolfi, Elisa Oltra, Tiziana Alberio, Mauro Fasano

**Affiliations:** Department of Science and High Technology, University of Insubria, Busto Arsizio (VA) 21052, Italy; Escuela de Doctorado, Universidad Católica de Valencia, San Vicente Mártir 46001, Spain; Department of Science and High Technology, University of Insubria, Como 22100, Italy; Department of Science and High Technology, University of Insubria, Busto Arsizio (VA) 21052, Italy; Department of Pathology, School of Medicine and Health Sciences, Universidad Católica de Valencia, San Vicente Mártir 46001, Spain; Department of Science and High Technology, University of Insubria, Busto Arsizio (VA) 21052, Italy; Department of Science and High Technology, University of Insubria, Busto Arsizio (VA) 21052, Italy

## Abstract

**Motivation:**

Drug repurposing offers a cost-effective and time-efficient strategy for identifying new therapeutic uses for existing medications, capitalizing on their known safety profiles and pharmacokinetics. We present an automated virtual screening pipeline using AutoDock Vina, a molecular docking software that predicts how small molecules bind to protein targets. This pipeline enhances the speed and accuracy of drug candidate identification by automating and parallelizing the docking process.

**Results:**

We developed and validated a fully automated virtual screening pipeline based on AutoDock Vina, enabling computational parallelization and random ligand positioning without relying on prior knowledge of biologically active protein domains. As a proof of concept, the pipeline was applied to the “serotonin and anxiety” pathway. Docking results were compared with known drug-target interactions, demonstrating the ability of the pipeline to reliably identify compounds interacting with serotonin receptors. This case study confirms the pipeline’s effectiveness in supporting drug repurposing by identifying promising candidates for further experimental validation.

**Availability and implementation:**

The AutoDock Vina automation pipeline is freely available for noncommercial use at https://gitlab.com/la_sveva/pip2.0. It is compatible with Linux systems, and a Docker image is provided for ease of deployment and reproducibility. Researchers can easily integrate the pipeline into existing workflows, supporting broader adoption in virtual screening and drug repurposing projects.

## 1 Introduction

Despite substantial advances in fields such as computational biology and chemistry, which allow unprecedented simulations of biological processes and molecular interactions (Ekins *et al.* 2007, Paul *et al.* 2010), drug development remains a significant challenge, and many medical conditions still lack effective treatments (Ballard *et al.* 2020, Fetro and Scherman 2020, Sisignano *et al.* 2022, Reach 2024). Innovative strategies, as drug repurposing, that is, discovering new therapeutic applications for existing drugs (Ashburn and Thor 2004), have gained increasing attention in recent years (Pushpakom *et al.* 2018). Drug repurposing uses the established pharmacological profiles and known mechanisms of action of existing drugs (Pushpakom *et al.* 2018, Gns *et al.* 2019, Parvathaneni *et al.* 2019, Jourdan *et al.* 2020), offering several advantages over traditional drug discovery, such as lower costs, shorter development timelines, and reduced risks of failure compared to *de novo* drug design (Nosengo 2016, Mullard 2017, Cha and Erez 2022). Unlike novel compounds, repurposed drugs have generally undergone extensive preclinical and clinical tests, as well as post-marketing safety assessments (pharmacovigilance data), which support their safety profiles and accelerate regulatory approval processes (Ashburn and Thor 2004, Nosengo 2016). In this context, drug repurposing also applies to compounds already on the market but approved for different clinical indications.

Virtual screening (VS) is a widely used computational technique in drug discovery and molecular biology fields, valuable for identifying potential drug candidates or bioactive compounds through simulated interactions with biological targets (Walters *et al.* 1998, Drwal *et al.* 2014, Oliveira *et al.* 2023). By employing advanced computational algorithms and molecular modeling methods, VS accelerates the identification of promising drug candidates defined as molecules selected for their potential to bind effectively to specific targets and modulate biological pathways involved in disease (Ashburn and Thor 2004, Wilkinson and Pritchard 2015, Oliveira *et al.* 2023). This approach provides substantial advantages over traditional laboratory methods, significantly reducing both time and costs while enabling high-throughput screening of large compound datasets. As a consequence, VS enhances the likelihood of identifying compounds with optimal efficacy and reduced toxicity, making them viable candidates for further preclinical or clinical investigations.

Several pipelines have been proposed in recent years for automating docking-based VS (Mendez *et al.* 2019, Chen *et al.* 2020), yet many of them present limitations. In particular, they often rely on predefined binding pockets, thus restricting the discovery of unexpected interactions, or lack automated parallelization, which reduces throughput in large-scale experiments. Other tools do not integrate post-processing procedures for systematic detection of outliers or cross-validation against reference databases, which weakens their applicability in drug repurposing studies.

Our work addresses these gaps by presenting a fully automated pipeline that: (i) systematically scans the entire receptor surface without prior knowledge of binding sites, (ii) employs random ligand positioning and triplicate docking runs to reduce stochastic bias, (iii) integrates high-throughput parallelization using Redis, and (iv) introduces a robust candidate prioritization scheme based on complementary normalization strategies (Z-score and MinMax) coupled with cross-database validation.

In particular, the pipeline enhances the reliability of blind docking, traditionally prone to false positives due to the large search space, making it a more robust tool for large-scale screening when no prior information on binding pockets is available. These features position our pipeline as a flexible and reproducible framework for virtual screening, capable of highlighting both known and potentially novel receptor–ligand interactions.

## 2 System and methods

### 2.1 File formats

In this work, we referred to multiple file formats frequently encountered in molecular docking. For simplicity, detailed technical specifications are reported at the end of the Supplementary Materials, available as supplementary data at *Bioinformatics Advances* online.

### 2.2 Pipeline overview

**Figure 1.**
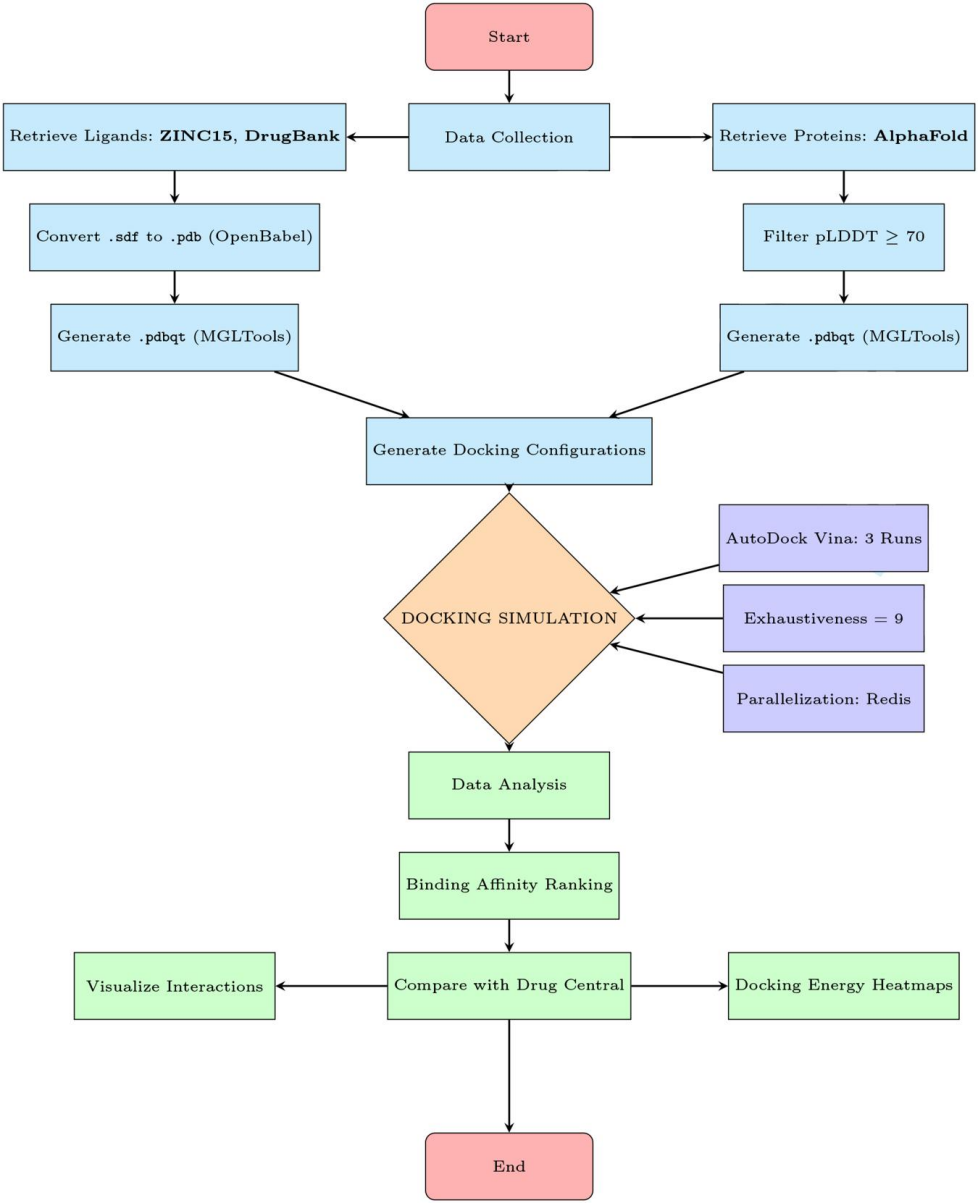
Schematic representation of the custom virtual screening workflow used for docking simulations with AutoDock Vina. The pipeline includes data collection from public databases, ligand and protein structure preparation, configuration generation, parallelized docking with AutoDock Vina, and downstream data analysis including visualization and comparison with drug interaction databases.

The pipeline has been designed to run on any Linux distribution, using Anaconda (2020) ana to create a flexible and efficient development environment able to support both Python 2 and Python 3, accommodating the dependencies of various packages. At its core, the pipeline relies on a curated list of target proteins representing the biological pathway network and a library of drug candidates formatted as .pdbqt files (Trott and Olson 2010, The Scripps Research Institute 2021), to perform VS with AutoDock Vina. Figure 1 provides a schematic representation of the pipeline, which includes:

1. Automated retrieval and preparation of ligands and receptors
2. Docking simulations with AutoDock Vina in triplicate
3. Parallelization via Redis (n.d.) to enable high-throughput processing
4. Automated post-processing and integration with DrugCentral reference database

### 2.3 Ligand preparation

Ligand structures were retrieved from the ZINC15 database (Irwin and Shoichet 2024) using a custom script designed to scrape all molecules relevant to our case study. Query filters were applied to retrieve only those classified as active in man and FDA-approved, thus prioritizing molecules with proven safety profiles and therapeutic potential. This selection strategy ensures a heterogeneous yet pharmacologically reliable library, including compounds already marketed for different clinical uses.

ZINC15 provides molecular structures in the .sdf format, which were then converted to .pdb using OpenBabel (Koes *et al.* 2024). OpenBabel infers or applies bond data based on interatomic distances and predefined covalent radii thresholds, ensuring chemically coherent .pdb files. Aromatic systems were identified by detecting delocalized π-electron configurations.

To ensure accurate electrostatic modeling and conformational flexibility during docking, an additional step was performed to enrich the molecular structures with partial atomic Gasteiger charges Gasteiger and Marsili (1980) and degrees of freedom of rotation. These data were then saved in .pdbqt files (The Scripps Research Institute 2021), the required format for AutoDock Vina.

### 2.4 Receptor preparation

For this study, we decided to focus on the serotonin and anxiety pathway (WP2141; WikiPathways 2025) (originally defined for *Mus musculus)*, Fig. 1, available as supplementary data at *Bioinformatics Advances* online. We first gathered the relevant proteins from this pathway and retrieved their UniProt identifiers (UniProt Consortium 2021), Table 1, available as supplementary data at *Bioinformatics Advances* online). To ensure translational relevance, the corresponding *Homo sapiens* orthologs were selected. Structural models have then been obtained from the AlphaFold database Jumper *et al.* (2021), which provides pLDDT score for each residue (ranging from 0 to 100). Higher pLDDT values indicate greater reliability in the local structure. To avoid including uncertain regions, residues with a pLDDT below 70 were removed from each protein model. The resulting curated structures were subsequently converted into .pdbqt format using MGLTools, which also assigned partial charges and allowed for the specification of flexible residues if needed.

**Table 1.** Additional drug–target pairs with previously known interaction, ranked by normalization method.

Z-score normalization	MinMax normalization
P28223–DB01104 Normalized value: −1.812	P28223–DB01104 Normalized value: 0.143
P28335–DB00472 Normalized value: −1.812	P28335–DB00472 Normalized value: 0.143
P08908–DB00715 Normalized value: −1.533	P08908–DB00715 Normalized value: 0.286

In this work, the receptor was treated as a rigid body during the docking simulations to maintain computational efficiency in a high-throughput screening context. Because no residues were designated as flexible, the pipeline could systematically explore the entire receptor surface for potential binding sites. While this strategy simplifies the workflow and reduces runtime, it does not account for potential side-chain rearrangements or induced-fit effects that may occur upon ligand binding. Future extensions of the pipeline will incorporate automated semi-flexible or fully flexible docking protocols, particularly in cases where conformational changes are known to be critical for ligand recognition. As an additional refinement step, such advanced simulations could be selectively applied only to the most promising hits identified in the initial rigid-docking screen, balancing thoroughness and computational cost.

### 2.5 Molecular docking simulations

All docking simulations were performed with AutoDock Vina (Trott and Olson 2010), using exhaustiveness  =9 to balance search thoroughness with computational cost. For each receptor–ligand pair, three independent docking runs were launched, each with a distinct random seed. These seeds were saved to ensure complete reproducibility. By initiating multiple searches from different random starting conditions, the pipeline reduces the risk of converging to local minima.

A Redis-based (Redis) queuing system was employed to orchestrate high-throughput workloads: the required simulations were enqueued once, and worker processes fetched tasks in parallel. Each docking job used nine CPU cores (reflecting the chosen exhaustiveness level). Depending on the available hardware, hundreds of jobs could be executed concurrently. AutoDock Vina produced .pdbqt files for each simulation, containing the predicted binding pose(s) and the corresponding energy scores. Upon completion of each job, the pipeline created a simple .done checkpoint file, allowing any unfinished tasks to be resumed automatically in the event of a crash or interruption.

#### 2.5.1 Data preprocessing and organization

After performing the docking simulations, a numerical matrix M was generated, in which each row corresponds to a specific receptor and each column represents a ligand. For each receptor–ligand pair, three independent docking runs were carried out; we selected the most favorable (i.e. lowest) binding energy and stored this value as $m_{ij}$. The resulting matrix $M=\{m_{ij}\}$ thus contains one binding-energy value per receptor–ligand pair, reflecting the best outcome (i.e. lowest energy) from the triplicate runs.

#### 2.5.2 Best candidates detection methodology

To systematically identify ligands with exceptionally low (favorable) binding energies, we applied two different normalization strategies: Z-score and MinMax. Both methods highlight outliers, but they do so under different assumptions, thereby ensuring robust detection of high-affinity receptor–ligand pairs.

### 2.6 Z-score normalization

First, we computed the mean and standard deviation of the binding energies for each receptor *i*. Denoting these as $\mu_{i}$ and $\sigma_{i}$, respectively, we calculated the *Z*-score for each pair (i,j) using:


(1) $$Z_{ij}=\frac{m_{ij}-\mu_{i}}{\sigma_{i}}.$$

This transformation measures how many standard deviations $m_{ij}$ lies below or above the receptor-specific mean $\mu_{i}$. We then considered receptor–ligand pairs as potential high-affinity “outliers” if


$$Z_{ij}\leq -1.541,$$

which corresponds to approximately the lowest 5% in a standard normal distribution.

### 2.7 MinMax normalization

In parallel, we applied MinMax scaling for each receptor *i*. Let


$$m_{i}^{\min}=\underset{j}{\min}(m_{ij}),\;m_{i}^{\max}=\underset{j}{\max}(m_{ij}),$$

be the minimum and maximum binding energies observed for receptor *i*. We defined the MinMax score as:


(2) $${\text{MinMax}}_{ij}=\frac{m_{ij}-m_{i}^{\min}}{m_{i}^{\max}-m_{i}^{\min}}.$$

We then isolated the bottom 5% of these scaled values, corresponding to the smallest (most negative) binding energies for each receptor. Unlike Z-score normalization, MinMax does not assume any particular distribution; it simply rescales the data into [0, 1], preserving relative ranks across the receptor-specific energy ranges.

#### 2.7.1 Candidate selection and cross-validation

Ligand-receptor pairs identified as belonging to the bottom 5% according to either Z-score or MinMax normalization criteria were initially flagged as high-affinity candidates. To further validate these interactions and assess their biological relevance, we cross-referenced the significant receptor-ligand pairs with the drug.target.interaction.tsv dataset from the DrugCentral database. Each pair was assigned a binary label:


$$\mathrm{Label}=\{\begin{array}{c} 1,\text{if}\;\text{matching}\;a\;\text{known}\;\text{drug}--\text{target}\;\text{entry},\\ 0,\text{otherwise}. \end{array}$$

This labeling allowed us to clearly distinguish between already documented interactions and potentially novel interactions that warrant experimental follow-up.

Additionally, to enhance the identification of particularly promising novel candidates (label = 0), we established a further selection criterion based on the lowest binding energy observed among known (label = 1) interactions. Specifically, the threshold for raw docking energies was set at −11.200 kcal/mol, corresponding to the strongest known documented interaction. Under this criterion, we identified 88 previously undocumented receptor-ligand pairs (label = 0) with binding energies even lower than this threshold.

Similarly, using MinMax normalization, we set the threshold at the lowest normalized value observed among known interactions, which was 0.071. Using this threshold, an additional 167 receptor-ligand pairs (label = 0) showed normalized values below this cutoff, marking them as promising high-affinity candidates for experimental validation.

By applying these complementary normalization strategies and thresholds derived from known interactions, we systematically prioritized both well-known and novel ligand-receptor pairs, thus significantly enhancing the robustness and biological relevance of the pipeline outcomes.

## 3 Results

### 3.1 Application to serotonin and anxiety pathway

The capabilities of the pipeline presented in this work were demonstrated by applying it to a real-world case study from the serotonin and anxiety pathway (WP2141 on WikiPathways; WikiPathways 2025). In total, 20 receptor structures and 4952 ligands were screened.

#### 3.1.1 Overall virtual screening execution

After data preprocessing, a 20×4952 matrix *M* of docking scores was obtained, where each matrix element $m_{ij}$ represents the most negative (best) binding energy across three runs for receptor *i* and ligand *j*. This triplicate approach, combined with random seeds for each run, minimized the risk of local minima and increased the robustness of the virtual screening.

#### 3.1.2 Quantitative summaries and outliers

Following the analysis procedure described in Section 2.5.1, two complementary normalization techniques, Z-score and MinMax, were employed to systematically identify ligand-receptor pairs with exceptionally low (favorable) binding energies. By combining these methods, we aimed to ensure a comprehensive and robust selection of high-affinity candidates.

Initially, we analyzed the dataset containing 20 receptors and 4952 ligands, resulting in a total of 99 040 ligand-receptor pairs. Among these, 477 pairs were already documented as true interactions in the DrugCentral database (label = 1). Using Z-score normalization, we identified a total of 4920 significant interactions (top 5% lowest energies). Within this subset, 21 of the previously known 477 pairs (4.40%) were flagged as extreme outliers. Similarly, using MinMax normalization, we also identified ∼4906 significant interactions, of which 22 of the known pairs (4.61%) were classified as extreme outliers.

**Figure 2.**
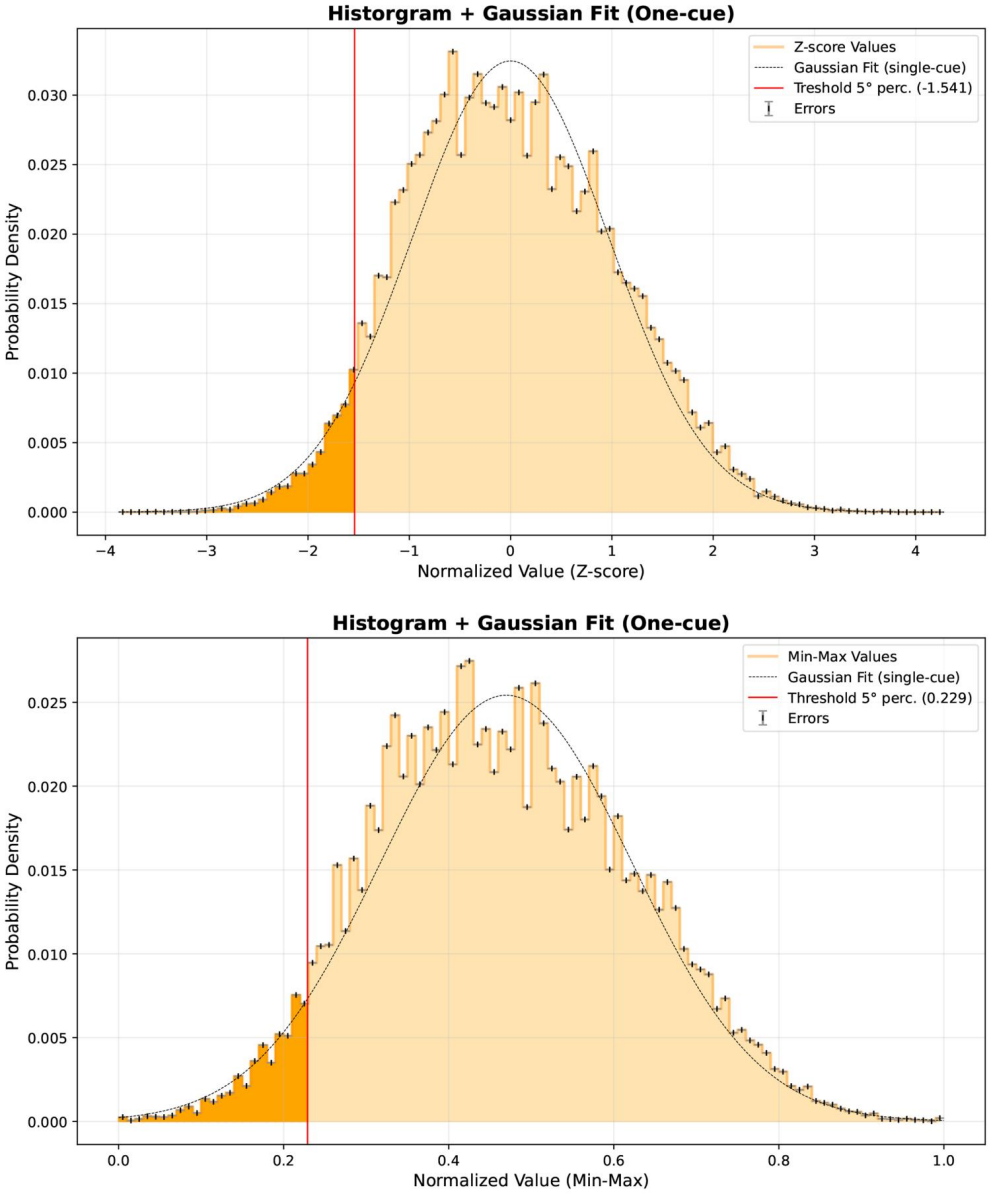
Comparison of threshold-based outlier detection using (a) Z-score and (b) MinMax normalization strategies on docking energies. Histograms include Gaussian fits and display the 5th percentile threshold. Errors are shown as vertical bars.

The distribution of normalized values obtained through the Z-score and MinMax methods are shown in Fig. 2a and b, respectively.

To further prioritize novel potential interactions (label = 0), we established thresholds based on the lowest binding energy observed among known interactions. Specifically, using raw docking energies, the lowest value among label = 1 pairs was found to be −11.200 kcal/mol. Under this threshold criterion, 88 previously undocumented receptor-ligand pairs showed energies even lower than −11.200 kcal/mol, highlighting them as promising candidates for further experimental evaluation. With the MinMax approach, the lowest normalized value among known pairs was 0.071, and 167 novel receptor-ligand pairs displayed MinMax scores below this value, marking them as similarly promising candidates for further investigation.

These selected receptor-ligand pairs constitute highly favorable interactions, demonstrating the capability of the pipeline in identifying not only previously known but also potentially novel therapeutic targets.

### 3.2 Normalization methods comparison

Among the receptor–drug pairs previously reported in the literature for their potential interaction, our pipeline identified 20 pairs as significant under both Z-score and MinMax normalization strategies, supporting the robustness of the method regardless of the normalization approach. The three common interactions with the lowest binding energies are reported in Table 1.

Regarding the differences between normalization methods, one pair, P28223–DB01012 (Z-score: −1.55, MinMax: 0.24), was identified only with Z-score normalization, while two pairs, P35348–DB00875 (Z-score: −1.50, MinMax: 0.22) and P35348–DB00502 (Z-score: −1.50, MinMax: 0.22), were detected exclusively using MinMax normalization (Table 2, available as supplementary data at *Bioinformatics Advances* online).

**Table 2.** Top drug–target pairs with no previously known interaction, ranked by normalization method.

Z-score normalization	MinMax normalization
Q13255–DB01142 Normalized value: −3.870	P28223–DB00476 Normalized value: 0.000
Q9BRC7–DB00934 Normalized value: −3.764	P28223–DB04896 Normalized value: 0.000
Q13255–DB02112 Normalized value: −3.651	P28335–DB00285 Normalized value: 0.000

These discrepancies highlight how the choice of normalization can influence the final selection of significant interactions.

The tables containing the significative couples both with and without previously reported interactions are listed in Table 3 and 4, available as supplementary data at *Bioinformatics Advances* online, respectively.

**Table 3.** Receptor–drug pairs identified as significant by the pipeline, including mapping, label (presence in DrugCentral), and the normalization method(s) that detected them.

UniProt ID	Gene	DrugBank ID	Drug Name	Label	Detected by
P28223	HTR2A	DB01104	Sertraline	1	Both
P28335	HTR2C	DB00472	Fluoxetine	1	Both
P08908	HTR1A	DB00715	Paroxetine	1	Both
Q13255	CRHR2	DB01142	Doxepin	0	Z-score
Q9BRC7	PLCD4	DB00934	Paroxetine	0	Z-score
Q13255	CRHR2	DB02112	Maprotiline	0	Z-score
P28223	HTR2A	DB00476	Duloxetine	0	MinMax
P28223	HTR2A	DB04896	Milnacipran	0	MinMax
P28335	HTR2C	DB00285	Venlafaxine	0	MinMax

**Table 4.** Comparison between existing docking-based virtual screening pipelines and the proposed automated pipeline.

Feature	Existing tools/pipelines	Our pipeline
Binding site definition	Often restricted to predefined or literature-based binding pockets, limiting the discovery of novel or unexpected interactions.	Systematic scanning of the entire receptor surface, enabling unbiased detection of potential binding sites without prior assumptions.
Docking runs	Single docking run per ligand–receptor pair, prone to stochastic variability.	Triplicate docking runs with distinct random seeds to minimize convergence to local minima and ensure reproducibility.
Receptor flexibility	Rigid docking in most cases; flexible docking rarely automated and often limited to selected residues.	Rigid docking by default for high-throughput efficiency; semi-flexible or fully flexible docking planned as a future extension through a dedicated accessory workflow.
Parallelization	Limited or manual parallelization; throughput depends on hardware configuration and user expertise.	Automated large-scale parallelization via Redis, enabling concurrent execution of hundreds of docking jobs with checkpoint recovery.
Post-processing and scoring	Docking scores often reported as raw binding energies, with no standardized normalization.	Dual outlier-detection strategy combining Z-score and MinMax normalization, ensuring robust prioritization across receptors with different energy distributions.
Cross-validation with external databases	Rarely included or only manual.	Automated comparison with DrugCentral reference dataset to distinguish known from novel drug–target interactions.
Output reproducibility	Partial, often lacking standardized workflow deployment.	Full reproducibility through open-source code, Docker container, and publicly available pipeline repository.

Among the evaluated receptor–drug pairs, Table 2 shows the top three most negative energy values among the couples with no previously detected interaction ranked by Z-score normalization:

To verify whether the pipeline successfully docked drugs in a potentially functional receptor site, a qualitative assessment was performed after identifying significant pairs. This involved generating images to visualize the PDB files of the receptors with their corresponding ligands, as well as the docking results produced by the pipeline. All the images are available in Supplementary Fig. 2, available as supplementary data at *Bioinformatics Advances* online.

To identify potentially interesting receptor–drug pairs among those labeled as 0 (i.e. not reported in the reference database), the lowest value observed among all known interacting pairs (1) was used as a threshold. (see Section 2.7.1).

For the analysis based on raw docking energies, the minimum value among the pairs labeled as 1 was −11.200 kcal/mol (Z-score: −2.08, MinMax: 0.13), corresponding to the pair (P28335, DB01126). Based on this threshold, 88 pairs labeled with 0 showed docking energies lower than −11.200 kcal/mol (Z-score: −2.08, MinMax: 0.13), and were therefore considered as high-priority candidates for further investigation.

Similarly, for the Min-Max normalization approach, the lowest normalized value among pairs labeled as 1 was 0.071, corresponding to the pair (P08908, DB00696). A total of 167 pairs showed normalized values below this threshold, representing additional promising candidates for experimental validation.

Thus, by using the known interactions (label = 1) as a reference threshold, the pipeline identified 88 significant receptor–ligand pairs under the Z-score method, and 167 pairs under the MinMax method, all of which were not previously reported and may represent novel findings of pharmacological relevance.

This threshold-based selection strategy allowed a focused reduction of the number of 0 pairs to be tested, prioritizing those with energy values comparable to or more extreme than known active pairs.

Although both normalization methods aim to highlight the most promising ligand–receptor pairs by focusing on low (favorable) binding energy values, they are based on fundamentally different statistical assumptions, which can lead to subtle but relevant differences in candidate selection.

The normalization of the Z-score evaluates each value in relation to the mean and variability of the energy distribution of the receptor. This makes it particularly suitable when the energy values for a given receptor span a wide range. It emphasizes how “unexpected” a given value is within the context of that receptor, and is sensitive to the presence of outliers or skewed distributions.

On the other hand, MinMax normalization is a purely rank-based approach that rescales all values between 0 and 1 using the receptor-specific minimum and maximum. It does not consider the shape of the distribution, but simply identifies the lowest-ranking values. This method ensures that the bottom 5% of each receptor energy range is always selected, even when the underlying distribution is narrow or uniform.

In practice, this means that Z-score may exclude values that are low in absolute terms but close to the mean of a receptor with a narrow energy range. Conversely, MinMax may include such values if they rank among the lowest, even if they are not statistically distant from the average. This difference explains the slight variation in the number and identity of selected outliers, and underscores the value of using both approaches in parallel to ensure comprehensive candidate detection.

### 3.3 Summary of significant receptor–ligand pairs

To facilitate biological interpretation and evaluate the practical relevance of the identified receptor-ligand interactions, we compiled a selection of the most significant pairs highlighted by our automated virtual screening pipeline. Each pair was annotated with its respective gene name (derived from the UniProt ID) and drug name (from the DrugBank ID). Additionally, pairs were classified according to their presence in the DrugCentral database, using labels indicating previously documented interactions (label = 1) or potentially novel associations (label = 0).

The top three receptor-ligand pairs identified by each normalization method represent the most energetically favorable interactions among all nonpreviously documented pairs. Specifically, using Z-score normalization, the top three significant novel pairs (label = 0).

Notably, several known high-affinity interactions were consistently identified by both normalization approaches, thus validating the robustness and accuracy of our pipeline. The detailed identification and prioritization of these receptor-ligand pairs demonstrate the capability of the pipeline to accurately recapitulate established therapeutic interactions and effectively pinpoint novel candidates. The highlighted novel interactions provide valuable starting points for further experimental investigation and potential therapeutic development as shown in Table 3.

## 4 Discussion

To evaluate the relevance of the virtual screening results, we focused on known drug–target relationships retrieved through our automated pipeline based on AutoDock Vina. This approach allowed us to efficiently identify high-affinity interactions involving clinically relevant compounds, paving the way for a pharmacological assessment of the findings.

A unique strength of our pipeline compared to existing workflows lies in its combination of unbiased receptor surface exploration, stochastic docking in triplicate, dual normalization outlier detection, and systematic cross-referencing with curated databases. This integrative design not only reproduces established interactions but also expands the search space toward novel receptor–ligand pairs that are not captured by traditional pipelines. The capability to operate without predefined binding sites, together with reproducible large-scale parallelization, makes our workflow distinctively suited for virtual screening applications.

Nevertheless, certain limitations must be acknowledged. In this study, receptors were treated as rigid bodies to maintain computational efficiency in a high-throughput context. While this simplification enables large-scale screening, it does not account for side-chain rearrangements or induced-fit effects that may occur upon ligand binding. Implementing semi-flexible or fully flexible docking protocols would require an additional dedicated workflow to identify, for each receptor, which residues should be defined as flexible and how this flexibility should be parameterized. This extension represents a promising future direction of our research, in which flexible docking could be selectively applied to top-ranked hits to balance accuracy and computational cost.

Another crucial step for future development is experimental validation. Although our computational results were supported by the recovery of several clinically established drug–target pairs, the novel interactions identified remain predictive hypotheses. Confirming their biological relevance will require in vitro or in vivo experiments, not only within the serotonergic system used as proof of concept here, but also across other therapeutic areas. We explicitly consider such validation the most important follow-up to strengthen the translational impact of our findings.

The main differences between existing docking-based workflows and our pipeline, highlighting the distinctive features that address current methodological limitations are depicted in Table 4.

As for the strengths of this work, both Z-score and MinMax normalization successfully brought to the fore receptor–drug interactions with unusually low docking energies (generally below the 5th percentile). By centering around the receptor-specific mean and scaling by standard deviation, Z-score normalization identifies outliers within the distribution of each individual receptor, as shown in Fig. 2a; this is particularly helpful when certain receptors display broad energy ranges due to intrinsic flexibility or docking constraints. In contrast, MinMax normalization provides an absolute rank-based perspective, as shown in Fig. 2b, ensuring that the bottom 5% are flagged regardless of the underlying distribution. The agreement between these two methods—in detecting many of the same clinically relevant pairs—demonstrates that our outlier-based approach is robust to the choice of normalization.

From a bioinformatics standpoint, the presence of well-characterized receptor–drug combinations among the top-ranked hits serves as a critical internal validation. These known pairs confirm that the automated workflow reliably captures genuine high-affinity interactions rather than random or trivial matches. Additionally, cross-referencing with external databases (e.g. labeling a match as 1 if found in DrugCentral) further corroborates these results. Such external validation not only strengthens confidence in the protocol but also highlights the potential of this pipeline to uncover novel, clinically relevant targets that may not yet be documented.

Because the pipeline accurately identified receptor–drug pairs already in clinical use, it implies a strong capacity to pinpoint new, high-affinity candidates for experimental follow-up. This synergy between established and potentially novel interactions is precisely what makes outlier-based ranking powerful: it provides a prioritized list of ligands whose predicted binding energies are consistently favorable. Future work may involve in vitro/in vivo testing of unverified hits—especially those flagged with no current DrugCentral entry (labeled 0)—to assess their therapeutic potential and expand the known pharmacological repertoire for these receptors.

In summary, both Z-score and MinMax normalization approaches effectively singled out meaningful receptor–drug interactions for clinically relevant targets. The overlap with validated clinical usage demonstrates the reliability and applicability of our docking-based pipeline. By integrating unbiased docking, robust normalization, and external validation, this methodology represents a reproducible and extensible framework for drug repurposing studies.

## Supplementary Material

vbaf267_Supplementary_Data

## Data Availability

The AutoDock Vina automation pipeline is freely available for noncommercial use at https://gitlab.com/la_sveva/pip2.0.
