# Supplementary material for: Unveiling novel drug-target couples: an empowered automated pipeline for enhanced virtual screening using AutoDock Vina
Source: Bioinform Adv. 2025 Nov 12;5(1):vbaf267. doi: 10.1093/bioadv/vbaf267 (PMC12699991; doi:10.1093/bioadv/vbaf267)
Supplement: vbaf267_Supplementary_Data [file vbaf267_supplementary_data.pdf]

Supplementary Materials

Input Nodes

Table 1s. List of UniProt IDs and their corresponding gene names involved in the serotonin and anxiety pathway. These genes encode proteins that play key roles in serotonin regulation and anxiety-related processes.

UniProt ID	Gene Names
O00418	EEF2K
P01100	FOS, G0S7
P01189	POMC
P05771	PRKCB, PKCB, PRKCB1
P08567	PLEK, P47
P08908	HTR1A, ADRB2RL1, ADRBRL1
P13639	EEF2, EF2
P14867	GABRA1
P28223	HTR2A, HTRA2
P28335	HTR2C, HTR1C
P35348	ADRA1A, ADRA1C
Q06787	FMR1
Q08209	PPP3CA, CALNA, CAN
Q13225	GRM1, GPCR1A, MGLUR1
Q13324	CRHR2, CRF2R, CRH2R
Q13554	CAM2KB, CAM2, CAMK2, CAMKB
Q7LC44	ARC, KIAA0278
Q9BRC7	PLCD4

Previously Reported Interactions Identified as Significative from the Pipeline

Table 2s. Comparison of previously detected interaction of significant drug–receptor interactions detected using Z-Score and MinMax Normalization.

(a) Z-Score Normalization

Receptor	Drug	Energy (KJ/mol)	Score
P28223	DB01012	-10.2	-1.548
P28223	DB01184	-10.3	-1.601
P28335	DB00696	-10.6	-1.764
P28335	DB01029	-10.5	-1.711
P28335	DB00358	-10.3	-1.604
P28335	DB01126	-11.2	-2.084
P28335	DB01349	-11.2	-2.084
P28335	DB01339	-10.2	-1.551
P28335	DB01184	-10.3	-1.604
P28335	DB01259	-10.5	-1.711
P28335	DB07145	-10.4	-1.657
P08908	DB00696	-10.6	-2.582
P08908	DB00224	-9.6	-1.947
P08908	DB01339	-9.3	-1.757
P08908	DB07519	-9.2	-1.693
P35348	DB01459	-8.7	-1.733
P35348	DB00266	-8.6	-1.657
P35348	DB00498	-8.6	-1.657
P35348	DB01261	-9.1	-2.038
P14867	DB01678	-7.9	-1.654
P14867	DB00845	-8.5	-2.127

(b) MinMax Normalization

Receptor	Drug	Energy (KJ/mol)	Score
P28223	DB01184	-10.3	0.228
P28335	DB00696	-10.6	0.179
P28335	DB01029	-10.5	0.187
P28335	DB00358	-10.3	0.205
P28335	DB01126	-11.2	0.125
P28335	DB01349	-11.2	0.125
P28335	DB01339	-10.2	0.214
P28335	DB01184	-10.3	0.205
P28335	DB01259	-10.5	0.187
P28335	DB07145	-10.4	0.196
P08908	DB00696	-10.6	0.071
P08908	DB00224	-9.6	0.172
P08908	DB01339	-9.3	0.202
P08908	DB07519	-9.2	0.212
P35348	DB01459	-8.7	0.191
P35348	DB00266	-8.6	0.202
P35348	DB00875	-8.4	0.225
P35348	DB00498	-8.6	0.202
P35348	DB00502	-8.4	0.225
P35348	DB01261	-9.1	0.146
P14867	DB01678	-7.9	0.219
P14867	DB00845	-8.5	0.146

Case Study

The *serotonin and anxiety pathway* (Figure 1s, WikiPathways WP2141) was selected as the case study for our proof-of-concept virtual screening pipeline because it involves a manageable number of proteins, allowing computational resources to focus on a well-defined set of molecular targets. Moreover, several established drugs target serotonin receptors in this pathway, with well-characterized binding sites. This background information makes it possible to compare computational docking results against existing experimental data, thereby providing a robust assessment of the pipeline's ability to identify active ligands and predict relevant molecular interactions. Overall, this pathway offers a balanced combination of biological complexity and computational accessibility, making it an optimal model for evaluating the proposed methodology.

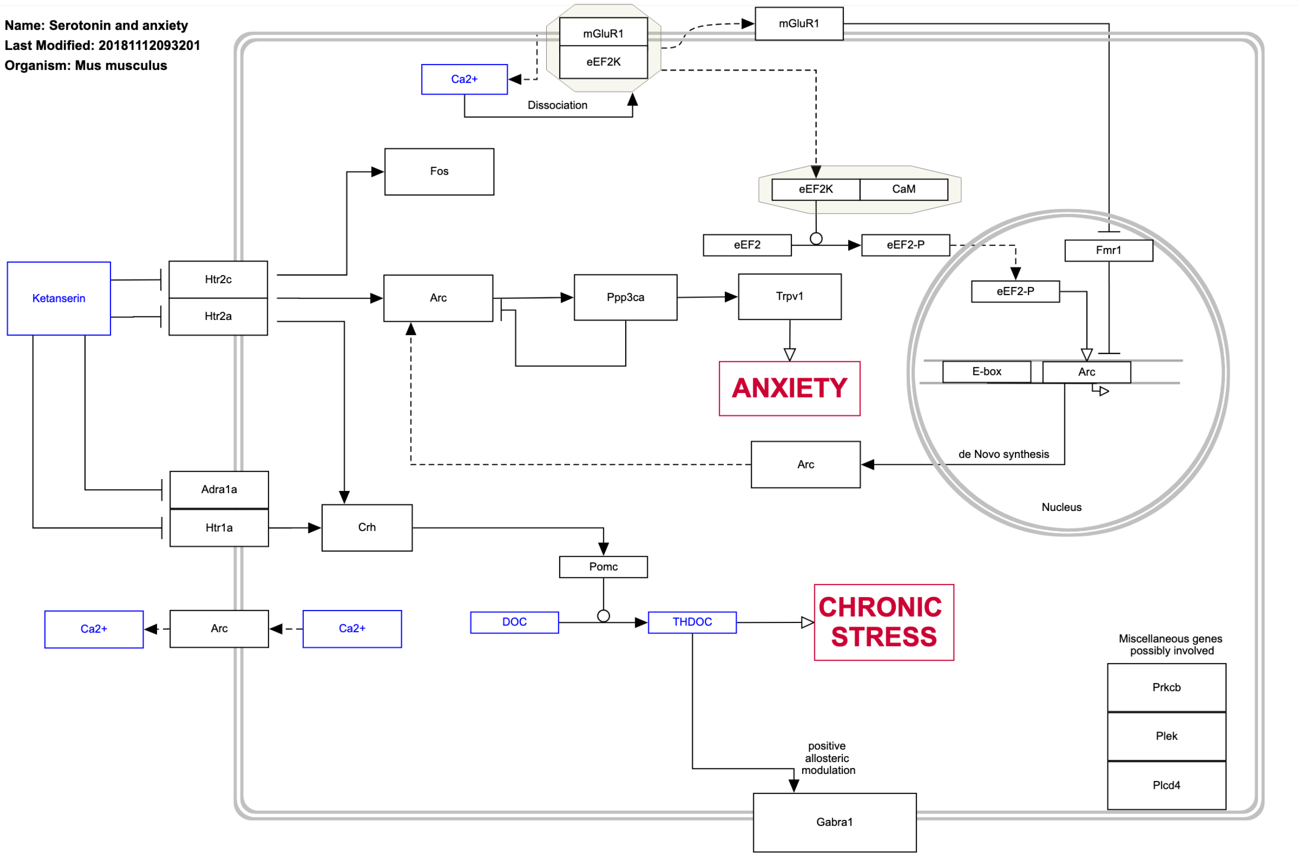

Figure 1s. The serotonin and anxiety pathway (WikiPathways WP2141). This pathway serves as a positive-control study.

Docking Results Visualization

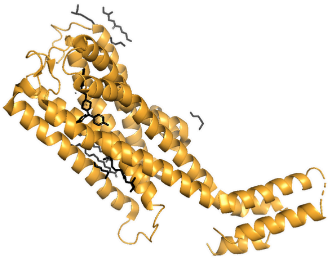

(a) 6BQH-P28335
Crystal structure of the HTR2C in complex with ritanserin provided by PDB.

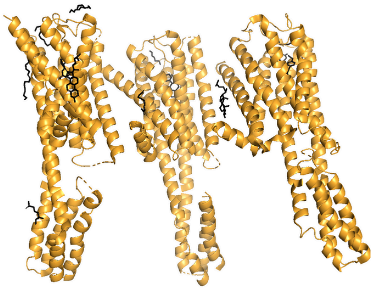

(b) 6WH4-P28223
Crystal structure of HTR2A with inverse agonist provided by PDB.

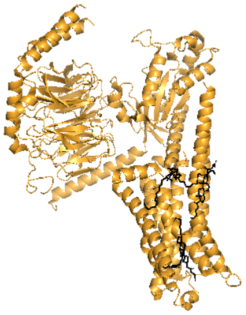

(c) 8FYX-P08908
Buspirone-bound serotonin 1A receptor-Gi1 protein complex provided by PDB.

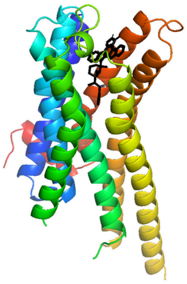

(d) P28223-DB06595
Docking pose of Midostaurin (DB06595) within the 5-HT2A receptor.

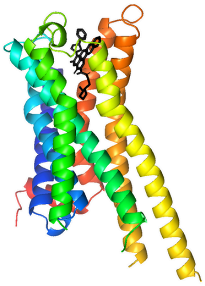

(e) P28223-DB06638
Docking pose of Quarfloxin (DB06638) within the 5-HT2A receptor.

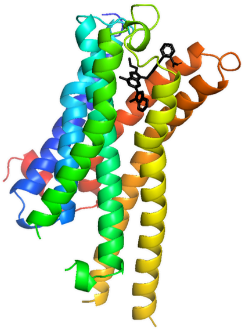

(f) P28335-DB00966
Docking pose of Telmisartan (DB00966) within the 5-HT2C receptor.

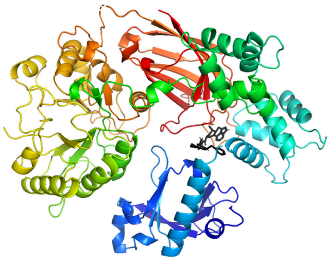

(g) Q8BRC7-DB00696
Docking pose of Ergotamine (DB00696) within the PLCD4 protein.

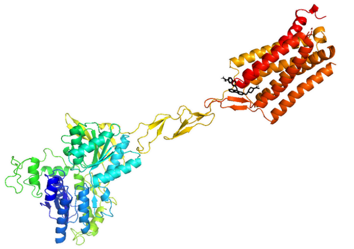

(h) Q13255-DB2112
Docking pose of ZK-806450 (DB2112) within mGluR1 (Q13255).

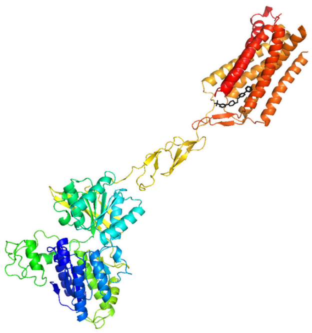

(i) Q13255-DB05454
Docking pose of Paliroden (DB05454) within mGluR1 (Q13255).

Figure 2s. Top: Crystallographic structures with known ligands (PDB IDs: 6BQH, 6WH4, 8FYX). Middle and bottom: top docking poses selected via Z-score and MinMax normalization.

Detection of Label-1 Receptor–Ligand Pairs Based on Binding Energy and Identification of Subthreshold Label-0 Candidates

Table 3s. List of receptor–ligand pairs with binding energy values corresponding to known true interactions (label = 1), as recorded in the reference database. Each row represents a receptor and its associated ligands, with respective predicted binding energy values and Z-score normalization. The lowest binding energy among all true positive pairs was −11.200 kcal/mol (pair: `P28335-DB01126`). A total of 88 receptor–ligand pairs not included in the database (label = 0) showed energy values lower than this threshold and were saved to the file `0s_below_min1energy.txt` for further analysis. Values are formatted as: `DBID: Binding Energy (kcal/mol) (Z-score)`.

Receptor	Ligands (DBID: Binding Energy (kcal/mol) (Z-score))
P28223	DB06638: -12.9 kcal/mol (-2.629), DB06925: -11.7 kcal/mol (-1.635), DB05984: -11.5 kcal/mol (-1.495), DB02323: -11.5 kcal/mol (-1.482), DB04477: -11.5 kcal/mol (-1.470), DB00941: -11.3 kcal/mol (-1.321), DB05038: -11.3 kcal/mol (-1.321), DB02112: -12.6 kcal/mol (-2.537), DB05450: -11.7 kcal/mol (-1.661), DB03067: -11.4 kcal/mol (-1.408), DB02258: -11.3 kcal/mol (-1.334), DB01419: -11.6 kcal/mol (-1.585), DB07041: -12.0 kcal/mol (-2.141), DB06555: -11.3 kcal/mol (-1.360), DB05678: -11.7 kcal/mol (-1.674), DB04881: -11.7 kcal/mol (-1.686), DB02269: -11.5 kcal/mol (-1.457), DB05075: -11.9 kcal/mol (-2.061), DB01879: -11.6 kcal/mol (-1.598), DB07307: -11.4 kcal/mol (-1.421), DB01897: -12.0 kcal/mol (-2.102), DB06666: -11.5 kcal/mol (-1.444), DB06896: -11.4 kcal/mol (-1.395), DB07514: -11.5 kcal/mol (-1.457), DB04868: -11.8 kcal/mol (-1.981), DB02741: -12.2 kcal/mol (-2.282), DB01988: -12.4 kcal/mol (-2.424), DB03231: -11.3 kcal/mol (-1.308), DB01761: -11.8 kcal/mol (-2.020), DB04852: -12.5 kcal/mol (-2.488), DB04727: -12.0 kcal/mol (-2.102), DB06595: -12.9 kcal/mol (-2.854), DB04689: -11.6 kcal/mol (-1.611), DB06630: -12.0 kcal/mol (-2.089), DB02226: -11.9 kcal/mol (-2.048), DB00210: -11.7 kcal/mol (-1.648), DB03331: -11.6 kcal/mol (-1.624), DB05454: -11.4 kcal/mol (-1.434)
P28335	DB04016: -11.3 kcal/mol (-1.457), DB06925: -12.0 kcal/mol (-2.237), DB04289: -11.7 kcal/mol (-1.791), DB05984: -11.5 kcal/mol (-1.648), DB00941: -11.4 kcal/mol (-1.567), DB02112: -12.5 kcal/mol (-2.608), DB00966: -12.6 kcal/mol (-2.689), DB06435: -11.5 kcal/mol (-1.661), DB04708: -11.7 kcal/mol (-1.791), DB06624: -11.3 kcal/mol (-1.484), DB02258: -11.8 kcal/mol (-1.866), DB03466: -11.4 kcal/mol (-1.580), DB02341: -11.3 kcal/mol (-1.470), DB02241: -11.4 kcal/mol (-1.567), DB06555: -11.6 kcal/mol (-1.732), DB04881: -11.3 kcal/mol (-1.470), DB02269: -11.5 kcal/mol (-1.661), DB00872: -12.3 kcal/mol (-2.437), DB07528: -11.3 kcal/mol (-1.470), DB04875: -11.4 kcal/mol (-1.567), DB01897: -11.8 kcal/mol (-1.866), DB06666: -11.3 kcal/mol (-1.484), DB04868: -11.9 kcal/mol (-1.949), DB02741: -12.0 kcal/mol (-2.237), DB06962: -11.5 kcal/mol (-1.648), DB04888: -11.3 kcal/mol (-1.470), DB07189: -11.5 kcal/mol (-1.648), DB04872: -11.5 kcal/mol (-1.648), DB01988: -11.9 kcal/mol (-1.986), DB01761: -11.6 kcal/mol (-1.732), DB04852: -12.1 kcal/mol (-2.310), DB06212: -11.7 kcal/mol (-1.791), DB04727: -11.3 kcal/mol (-1.457), DB06630: -12.4 kcal/mol (-2.520), DB03768: -11.3 kcal/mol (-1.457), DB05454: -11.5 kcal/mol (-1.661)
P21964	DB04016: -11.3 kcal/mol (-1.483), DB03005: -11.3 kcal/mol (-1.483), DB01419: -11.6 kcal/mol (-1.638), DB00320: -11.7 kcal/mol (-1.721), DB04868: -11.6 kcal/mol (-1.638), DB02226: -11.3 kcal/mol (-1.483)
Q9BRC7	DB00696: -12.0 kcal/mol (-2.179), DB00320: -11.6 kcal/mol (-1.632)
P05771	DB06630: -11.6 kcal/mol (-1.585)
P08908	DB04016: -11.3 kcal/mol (-1.185)
Q8NER1	DB04739: -11.3 kcal/mol (-1.276)
Q13255	DB02112: -11.4 kcal/mol (-1.483), DB06393: -11.3 kcal/mol (-1.414), DB05454: -11.7 kcal/mol (-1.641)

Detection of Label-1 Receptor–Ligand Pairs and Subthreshold Label-0 Candidates

Table 4s. List of receptor–ligand pairs with binding energy values and corresponding MinMax-normalized values. Each row represents a receptor and its associated ligands. The lowest MinMax value among all true positive pairs was 0.071 (pair: `P08908-DB00696`). A total of 167 receptor–ligand pairs not present in the database (label = 0) showed MinMax values lower than this threshold and were saved to `0s_below_min1_minmax.txt` for further analysis. Values are formatted as: `DBID: Binding Energy (kcal/mol) (MinMax)`.

**Receptor**	**Ligands (DBID: Binding Energy (kcal/mol) (MinMax))**
P08908	DB00599: -5.2 kcal/mol (0.616), DB00479: -6.6 kcal/mol (0.475), DB01047: -8.9 kcal/mol (0.242), DB01012: -8.1 kcal/mol (0.323), DB00458: -6.9 kcal/mol (0.444), DB03726: -6.2 kcal/mol (0.515), DB00715: -8.4 kcal/mol (0.293), DB00899: -6.3 kcal/mol (0.505), DB07573: -6.0 kcal/mol (0.535), DB01355: -6.0 kcal/mol (0.535), DB01170: -5.6 kcal/mol (0.576), DB00604: -7.8 kcal/mol (0.354), DB00265: -5.6 kcal/mol (0.576), DB00447: -7.4 kcal/mol (0.394), DB00377: -8.6 kcal/mol (0.273), DB00696: -10.6 kcal/mol (0.071)
P14867	DB00718: -5.5 kcal/mol (0.512), DB00372: -5.8 kcal/mol (0.476), DB01125: -6.7 kcal/mol (0.366), DB00260: -3.7 kcal/mol (0.732), DB01291: -5.1 kcal/mol (0.561), DB00246: -7.6 kcal/mol (0.256), DB00500: -6.1 kcal/mol (0.439), DB00490: -6.9 kcal/mol (0.341), DB01639: -4.9 kcal/mol (0.585), DB00430: -7.2 kcal/mol (0.305)
P21964	DB00527: -7.1 kcal/mol (0.451), DB00887: -7.6 kcal/mol (0.402), DB00346: -8.0 kcal/mol (0.363)
P28223	DB00599: -6.2 kcal/mol (0.588), DB01716: -5.6 kcal/mol (0.640), DB01475: -7.9 kcal/mol (0.439), DB00479: -7.0 kcal/mol (0.518), DB01012: -10.2 kcal/mol (0.237), DB00458: -7.7 kcal/mol (0.456), DB03726: -6.9 kcal/mol (0.526), DB00715: -8.9 kcal/mol (0.351), DB00929: -7.0 kcal/mol (0.518)
P35348	DB01716: -5.8 kcal/mol (0.517), DB00623: -6.9 kcal/mol (0.393), DB00458: -7.7 kcal/mol (0.303), DB00715: -8.2 kcal/mol (0.247), DB00899: -6.3 kcal/mol (0.461), DB01244: -7.0 kcal/mol (0.382), DB07573: -6.5 kcal/mol (0.438), DB00486: -7.8 kcal/mol (0.292), DB01355: -6.8 kcal/mol (0.404), DB01170: -5.7 kcal/mol (0.528), DB00604: -6.8 kcal/mol (0.404), DB00913: -6.9 kcal/mol (0.393), DB00447: -6.6 kcal/mol (0.427), DB00228: -5.5 kcal/mol (0.551), DB00990: -7.8 kcal/mol (0.292), ... DB00878: -7.6 kcal/mol (0.315)
Q08209	DB00710: -4.6 kcal/mol (0.632)
Q13255	DB01065: -6.2 kcal/mol (0.534)
Q13554	DB06637: -3.8 kcal/mol (0.747), DB01408: -6.5 kcal/mol (0.463)
Q8NER1	DB00481: -8.6 kcal/mol (0.273)

File Formats

In this work, we referred to multiple file formats frequently encountered in molecular docking. For simplicity, detailed technical specifications were not discussed in the main text. This section therefore provides an overview of the most common file formats and their typical usage in virtual screening pipelines.

`.sdf` *(Structure Data File)*

The `.sdf` format is widely used in cheminformatics to store molecular structure data, commonly employed for both virtual screening and molecular modeling. It represents structural and chemical information for each molecule, including atoms, bonds, and selected descriptors.

A single `.sdf` file can contain multiple entries (i.e., multiple molecules), each comprising:

Atomic Coordinates: three-dimensional coordinates (x, y, z) of each atom, facilitating molecular visualization and manipulation.

Atom and Bond Information: details about the connectivity of atoms (e.g., single, double, triple, aromatic bonds), enabling software to reconstruct the molecular graph.

Molecular Properties: optional descriptors, such as molecular weight, chemical formula, logP, hydrogen-bond donor/acceptor counts, and polar surface area. These properties help assess the drug-likeness of a compound and predict its behavior in biological systems.

Partial Charges: when available, per-atom partial charges can also be included, relevant for modeling electrostatic interactions.

Usage in Molecular Docking.

In docking workflows, `.sdf` files typically store small-molecule ligands in three-dimensional conformations. Although `.sdf` does not inherently track rotatable bonds or torsional flexibility, external software tools (e.g., Open Babel, RDKit) can generate such information from the raw coordinates. As a result, `.sdf` files are convenient for:

- Storing structural and chemical data for extensive ligand libraries.
- Representing molecular descriptors essential for scoring and filtering compounds.
- Providing a standardized input format compatible with many cheminformatics platforms.

Despite its flexibility, `.sdf` often requires post-processing to incorporate detailed torsional and charge information crucial for accurate docking.

`.pdb` *(Protein Data Bank)*

The `.pdb` format is a cornerstone in structural biology, designed to capture the three-dimensional coordinates of macromolecules (proteins, nucleic acids) and any associated cofactors or ligands.

A typical `.pdb` file includes:

Atomic Coordinates: the spatial positions of all atoms, serving as a basis for molecular visualization and computational analyses.

Atom Types: each atom is identified by element type (C, N, O, etc.) and a unique index for reference within the structure.

Residue Information: proteins and nucleic acids are organized into residues, each labeled with a residue name and index.

Chain Information: macromolecules often comprise multiple chains (e.g., subunits of a protein complex). The `.pdb` file records which residues belong to which chain.

Secondary Structure Elements: annotations for alpha helices, beta sheets, loops, or other features.

Heteroatoms and Ligands: non-polypeptide entities, such as metal ions, small-molecule ligands, and solvent molecules.

Usage in Molecular Docking.

In docking studies, `.pdb` files typically represent the receptor structure (protein). Key reasons for using `.pdb` files include:

- Providing the three-dimensional reference frame for ligand binding.
- Detailing important structural and sequence-based information, such as residue identities and chain definitions.
- Ensuring compatibility with numerous modeling tools (e.g., PyMOL, AutoDock Vina).

However, `.pdb` files do not inherently store partial charges or explicit torsional-flexibility data, which must be assigned or parameterized using additional software prior to docking.

`.pdbqt` *(AutoDock Format)*

The `.pdbqt` format extends the `.pdb` structure file to include partial charges and torsional degrees of freedom, aligning with the requirements of AutoDock4 and AutoDock Vina.

A `.pdbqt` file typically contains:

Atomic Coordinates: similar to `.pdb`, but organized into records suitable for docking algorithms.

Atom Types: each atom is labeled with an AutoDock-specific atom type, incorporating both element and partial charge.

Partial Charges: atomic partial charges are critical for calculating electrostatic contributions to binding affinity.

Rotatable Bonds: the file specifies which bonds can rotate, allowing exploration of multiple ligand conformations.

Usage in Molecular Docking.

Because `.pdbqt` provides both the receptor and ligand with essential docking parameters (partial charges, flexibility information), it is the standard input for AutoDock-based simulations. The format enables:

- Systematic sampling of ligand conformations by rotating designated bonds.
- Calculation of interaction energies, including hydrogen bonding, hydrophobic effects, and electrostatic forces.
- Straightforward integration into automated screening pipelines, where large libraries of ligands are tested against a defined binding site.

By consolidating crucial docking-related data, `.pdbqt` facilitates efficient and reproducible virtual screening protocols.
